# Association of serum vitamin D levels with clinical severity and recovery duration in cervical dizziness

**DOI:** 10.1186/s12891-026-10119-1

**Published:** 2026-06-23

**Authors:** Yaser Said Cetin, Semra Agirbas

**Affiliations:** Department of Otorhinolaryngology, Faculty of Medicine, Van Yüzüncü Yıl University, Van, Turkey

**Keywords:** Cervical dizziness, Vitamin D deficiency, Neck pain, Proprioception, Musculoskeletal disorders, Postural stability

## Abstract

**Background:**

Cervical dizziness (CD) is associated with altered cervical musculoskeletal and proprioceptive input, leading to impaired sensorimotor integration and balance control. Vitamin D deficiency has been linked to musculoskeletal pain, muscle dysfunction, and impaired postural stability; however, its role in CD remains unclear.

**Methods:**

This prospective observational study included 210 patients diagnosed with CD. Serum 25-hydroxyvitamin D [25(OH)D] levels were measured at baseline, and participants were categorized into deficiency (< 20 ng/mL), insufficiency (20–29 ng/mL), and sufficiency (≥ 30 ng/mL) groups. All patients received a standardized multimodal cervical rehabilitation program, with vitamin D supplementation provided to the deficient and insufficient groups. Clinical outcomes were assessed using the Neck Disability Index (NDI), Dizziness Handicap Inventory (DHI), and Vertigo Dizziness Imbalance questionnaires (VDI-SS and VDI-QOL). Associations between vitamin D levels, baseline symptom severity, and recovery duration were analyzed using correlation, multivariable regression, receiver operating characteristic (ROC) analysis, and partial least squares discriminant analysis (PLS-DA).

**Results:**

Lower serum 25(OH)D levels were significantly associated with higher baseline NDI, DHI, VDI-SS, and VDI-QOL scores (all *p* < 0.001), indicating greater symptom severity and functional impairment. Serum 25(OH)D levels showed a strong inverse correlation with recovery duration (rho = -0.77, *p* < 0.001) and remained associated with a longer recovery duration in multivariable linear regression. ROC analysis demonstrated high discriminatory performance for delayed recovery (AUC = 0.937), with an exploratory optimal threshold of ≤ 17 ng/mL. In exploratory PLS-DA, serum 25(OH)D contributed more to group separation than did age, sex, or BMI.

**Conclusions:**

Lower serum vitamin D levels were associated with greater baseline clinical severity and longer recovery duration in patients with CD. These findings suggest that vitamin D status may be clinically relevant in the assessment of disease severity and recovery in this population. Further prospective controlled studies are required to clarify the clinical significance of this association.

**Supplementary Information:**

The online version contains supplementary material available at https://doi.org/10.1186/s12891-026-10119-1.

## Introduction

Cervical dizziness (CD) is defined as dizziness or balance disturbance associated with cervical pathology, and is typically exacerbated by head movement [1]. It is thought to arise from disturbed cervical proprioceptive input, whereby altered sensory signals from contracted cervical muscles may lead to disorientation and a subjective sense of imbalance. Balance depends on the coordinated interaction of the vestibular, visual, and proprioceptive systems, as well as postural and muscular control. Disturbances in these systems may manifest as imbalances, limited cervical range of motion, instability, and neck pain. Because no definitive diagnostic test exists, CD is generally considered a diagnosis of exclusion after other vestibular, neurological, and medical causes have been ruled out [1, 2].

Neck pain, muscle spasms, or fatigue may alter proprioception and balance control, whereas prolonged cervical muscle contraction may increase the sensitivity of cervical proprioceptors [3]. Inflammatory mediators released during muscle spasms sensitize the muscle spindles and contribute to CD. Because head movements worsen symptoms in most vestibular conditions, increased cervical muscle tension may result from and contribute to reduced head movement [3].

In recent years, the association between vitamin D deficiency and various musculoskeletal disorders has gained increasing interest in the literature. Vitamin D plays an important role in muscle contraction, calcium-phosphorus metabolism, inflammatory modulation, and neuromuscular transmission. Previous studies have demonstrated that low vitamin D levels are associated with widespread musculoskeletal pain, myalgia, neck and low back pain, reduced muscle strength, impaired balance, and an increased risk of falling [4, 5]. In addition, previous studies have reported an association between vitamin D deficiency and certain vestibular disorders, particularly benign paroxysmal positional vertigo (BPPV), suggesting that low vitamin D levels may contribute to disease occurrence, persistence, and recurrence in these patients [6, 7]. However, despite these established musculoskeletal effects and the growing evidence from vestibular disorders such as BPPV, the role of vitamin D in CD and cervical-related balance disorders remains unclear. Therefore, this study aimed to investigate the association between serum 25(OH)D levels, baseline clinical severity, and recovery duration in patients with CD, while additionally describing available demographic, anthropometric, occupational, lifestyle, and seasonal characteristics relevant to cervical spine-related symptoms.

## Materials and methods

### Study design and setting

This was a prospective, observational study. Ethical approval was obtained from the local ethics committee (decision no. 2024-07, dated February 28, 2024). Written informed consent was obtained from all participants before enrollment. This study was conducted and reported in accordance with the Strengthening the Reporting of Observational Studies in Epidemiology (STROBE) guidelines. Vitamin D supplementation was administered as part of routine clinical care based on baseline serum levels and was not assigned for research purposes.

### Participants

The study was conducted between February 2024 and January 2026 at the Otolaryngology (ENT) outpatient clinic of a tertiary referral hospital. Eligible patients presenting during the study period were consecutively screened according to predefined inclusion and exclusion criteria. Participants who met the eligibility criteria and agreed to participate were enrolled. Forty-seven otherwise eligible patients declined to participate, mainly because of their unwillingness to complete the questionnaires or undergo blood sampling. To reduce potential confounding factors and improve sample homogeneity, the study was restricted to young adults aged 18–40 years without systemic disease. This age range was selected because dizziness in older individuals is more likely to be influenced by age-related cardiovascular, neurological, metabolic, and degenerative musculoskeletal conditions, which could affect balance responses and make clinical interpretation of CD more difficult.

The inclusion criteria were as follows: diagnosis of CD accompanied by pain on cervical palpation, increased tension and tenderness of the cervical muscles, and clinically restricted cervical range of motion as assessed during physical examination. In addition, the participants had no prior history of canalith repositioning maneuvers for dizziness, no use of ototoxic medications, and normal hearing, as confirmed by audiological evaluation.

The exclusion criteria were BPPV, Ménière’s disease, migraine-associated vertigo, vertebrobasilar insufficiency, postural hypotension, a history of cervical trauma, and cervical pathologies identified on magnetic resonance imaging, such as spondylosis, disc herniation, fractures, dislocation, spondylolisthesis, and scoliosis. Patients with fibromyalgia, myofascial pain syndrome, neurological, rheumatological, or systemic diseases (e.g., multiple sclerosis, Parkinson’s disease, rheumatoid arthritis), vascular pathologies detected by vertebral or carotid artery Doppler ultrasonography, central nervous system disorders identified on cranial MRI (e.g., multiple sclerosis or degenerative diseases), psychiatric disorders or a history of depression, and metabolic conditions such as anemia, vitamin B12 deficiency, or folate deficiency were excluded from the study.

### Diagnostic assessment

Diagnostic evaluation and clinical tests were performed in the otorhinolaryngology clinic by a single ENT specialist. At the time of diagnostic assessment, the treating clinician was unaware of the participants’ serum vitamin D levels because blood tests were only requested after the clinical diagnosis had been established. Neurology consultation was obtained for all patients to exclude central neurological causes and other non-otologic etiologies of dizziness, and patients with vascular and central nervous system pathologies were excluded from the study. Inter-rater reliability was not assessed because the diagnostic evaluation and clinical tests were performed by a single ENT specialist. For diagnostic purposes, all participants underwent a detailed clinical evaluation and medical history, and the following specific tests were performed in patients presenting with balance complaints to support the diagnosis of CD. Head–Neck Differentiation Test: The clinician seated the patient on a swivel chair and stabilized the head while rotating the trunk to assess dizziness. The procedure was repeated with the head released. The presence of dizziness during trunk rotation with the head stabilized was considered supportive of a cervical contribution to symptoms [8]. Seated Cervical Torsion Test: With the patient’s head fixed, the trunk was rotated 90° to the right or left, and each position was maintained for 30 s. Eye movements were assessed and recorded using infrared video Frenzel goggles. Nystagmus observed with the head in a neutral position was compared with that observed during cervical rotation. The presence of nystagmus with a slow-phase velocity (SPV) exceeding 2°/s was considered positive [9, 10]. These tests were used as supportive clinical maneuvers rather than stand-alone definitive diagnostic tests, and the diagnosis of CD was made in the context of the overall clinical evaluation after exclusion of peripheral and central causes of dizziness [9, 10]. Restriction of cervical range of motion was assessed clinically during physical examination and was not quantified using formal goniometric measurements.

### Treatment approach

The treatment approach included a standardized multimodal program administered in collaboration with the Physiotherapy and Rehabilitation Unit. All patients received cervical manual therapy and a structured exercise program. This protocol was based on previous studies supporting multimodal conservative management, including manual therapy, exercise, proprioceptive training, and patient education, in patients with CD and neck pain [11]. During the treatment period, patients were followed up weekly at the Otolaryngology outpatient clinic. Patients were educated to avoid inappropriate neck positions and participated in a regular cervical exercise program. Exercises aimed at strengthening the deep cervical flexor muscles were initiated with chin-tuck exercises in the supine position. Proprioceptive exercises, including head repositioning and visual targeting using a laser pointer, were performed in conjunction with balance-training. Cervical mobilization and stretching exercises were incorporated into the program, including gentle stretching of the trapezius, levator scapulae, and splenii capitis muscles. A home exercise program was prescribed, with written exercise brochures provided to the patients, and exercises were recommended to be performed twice a day. In addition, the patients received ergonomic education covering working postures, mobile phone and tablet use, pillow height, and protection against cold and windy environments.

### Vitamin D assessment

*Serum 25-hydroxyvitamin D [25(OH)D] levels were measured at the baseline.* Vitamin D status was classified according to widely accepted clinical reference ranges as deficiency (< 20 ng/mL), insufficiency (20–29 ng/mL), or sufficiency (≥ 30 ng/mL). *Participants were divided into three groups based on their baseline serum 25(OH)D concentrations. In addition to the standard physical and ergonomic treatment protocol*,* patients in the vitamin D deficiency and insufficiency groups received oral cholecalciferol at a daily dose of 2*,*000 IU. The supplementation dose was selected according to the commonly accepted clinical practice for the management of vitamin D deficiency and insufficiency. Patients with sufficient vitamin D levels did not receive vitamin D supplementation and were treated with physical and ergonomic therapies.* At the eighth week, serum 25(OH)D levels were remeasured to assess whether vitamin D levels had normalized. *Seasonal variation and individual sun exposure were not quantified separately in the present study.*

### Outcome measures

Patients with neck pain and a diagnosis of CD were evaluated regularly at baseline and during follow-up visits throughout the study treatment period. The scales used to assess the pretreatment status and treatment response were as follows: All instruments used in this study were previously validated and demonstrated acceptable psychometric properties in the literature.

#### Neck Disability Index (NDI)

The NDI was used to assess neck pain–specific disability and was modified from the Oswestry Disability Index. It consists of 10 sections evaluating pain intensity, personal care, lifting, reading, headaches, concentration, work, driving, sleep, and recreational activities. Each section includes six response options and is scored from 0 (no disability) to 5 (total). Based on the total score, disability was classified as follows: 0–4, no disability; 5–14, mild disability; 15–24, moderate disability; 25–34, severe disability; and ≥ 35, complete disability. The NDI is a widely used instrument with established validity and reliability for assessing neck-related disability, and the validated Turkish version was used in the present study [12, 13].

#### Dizziness Handicap Inventory (DHI)

For each item, participants responded “yes” (4 points), “sometimes” (2 points), or “no” (0 points). The total score ranges from 0 to 100, with higher scores indicating greater perceived dizziness-related handicap. The DHI is a well-established and validated questionnaire for evaluating the functional, emotional, and physical impacts of dizziness, and the validated Turkish version was administered to the participants in the present study [14, 15].

#### Vertigo Dizziness Imbalance (VDI) questionnaire

The VDI consists of two subscales: the Symptom Scale (VDI-SS; 14 items) and the Quality of Life Scale (VDI-QOL; 22 items). Each item is scored from 0 to 5, and total scores are obtained by summing the item responses (total score range: 0–70 for the VDI-SS and 0-110 for the VDI-QOL). Higher scores indicate more severe symptoms and poorer quality of life. The VDI has been reported to be a valid and reliable instrument for assessing vertigo- and dizziness-related symptoms and quality of life, and the Turkish version has also demonstrated acceptable psychometric properties [16–18]. Recovery duration was defined as the number of weeks from treatment initiation to sustained clinical improvement based on improvement in dizziness-related symptoms at follow-up and stabilization of clinical assessment scores. For descriptive purposes, recovery was categorized as early (≤ 4 weeks) or delayed (≥ 5 weeks).

### Statistical analysis

Sample size estimation was conducted utilizing G*Power software version 3.1. Based on an effect size (Cohen’s d) of 0.50, a two-sided significance level of 0.05, and a statistical power of 80%, a minimum of 65 participants per group was deemed necessary. All statistical analyses were executed using IBM SPSS Statistics version 26.0. The distribution of continuous variables was evaluated using the Shapiro-Wilk test. Data that were normally distributed are presented as mean ± standard deviation, whereas data that were not normally distributed are expressed as the median with interquartile range (IQR). Comparative analyses among the three vitamin D groups were conducted utilizing one-way analysis of variance (ANOVA) for variables exhibiting normal distribution, and the Kruskal–Wallis test for those not normally distributed. Upon identifying overall significance, post hoc pairwise comparisons were executed with appropriate adjustments for multiple testing. Categorical variables were assessed using the chi-square test. The relationships between serum 25-hydroxyvitamin D [25(OH)D] levels, clinical scale scores, and recovery duration were examined through Spearman’s correlation analysis. To evaluate the association between serum 25(OH)D levels and recovery duration, multivariable linear regression was employed, adjusting for age and sex. Furthermore, a multivariable binary logistic regression model was applied to assess delayed recovery, adjusting for demographic, anthropometric, occupational, and lifestyle covariates, including age, sex, BMI, occupation category, occupational activity level, work environment, prolonged desk/screen-related neck-flexion exposure, and exercise level. The season of blood sampling was descriptively evaluated across vitamin D categories.The receiver operating characteristic (ROC) curve analysis was employed to assess the discriminatory capability of serum 25(OH)D levels in predicting delayed recovery. The area under the curve (AUC), along with 95% confidence intervals, was computed, and the optimal cutoff value was identified using the Youden index, with corresponding sensitivity and specificity values reported. For exploratory multivariate classification, partial least squares discriminant analysis (PLS-DA) was conducted using serum 25(OH)D level, age, sex, and BMI to evaluate their combined influence on early and delayed recovery patterns. Variable importance in projection (VIP) scores were utilized to assess the relative contributions of each variable. All statistical tests were two-tailed, and a p-value of < 0.05 was considered statistically significant. Adjusted significance thresholds were applied where necessary to account for multiple comparisons.

## Results

The study encompassed 210 patients diagnosed with CD, who were categorized based on their baseline serum 25-hydroxyvitamin D [25(OH)D] levels: those with vitamin D deficiency (< 20 ng/mL, *n* = 69), vitamin D insufficiency (20–29 ng/mL, *n* = 69), and vitamin D sufficiency (≥ 30 ng/mL, *n* = 72). Table 1 provides a comprehensive summary of baseline demographic, anthropometric, occupational, lifestyle, and seasonal characteristics. The groups did not differ significantly in terms of age, sex distribution, body mass index (BMI), occupation category, occupational activity level, work environment, prolonged desk/screen-related neck-flexion exposure (≥ 6 h/day), exercise level, or the season during which blood samples were collected (all *p* > 0.05).

**Table 1:** Baseline demographic, anthropometric, occupational, lifestyle, and seasonal characteristics according to serum 25(OH)D categories

Variable	< 20 ng/mL (*n* = 69)	20–29 ng/mL (*n* = 69)	≥ 30 ng/mL (*n* = 72)	*P* value
Age, years	28 (26–33)	30 (28–31)	30 (27–33)	0.244
Sex - Female	47 (68.1)	48 (69.6)	52 (72.2)	0.864
Male	22 (31.9)	21 (30.4)	20 (27.8)	
BMI, kg/m²	23.59 (20.74–26.91)	24.83 (22.36–28.15)	25.54 (20.80-27.88)	0.159
Occupation category				
Student	6 (8.7)	10 (14.5)	10 (13.9)	0.491
Office/desk worker	27 (39.1)	31 (44.9)	27 (37.5)	
Healthcare worker	6 (8.7)	4 (5.8)	9 (12.5)	
Manual/physical worker	15 (21.7)	14 (20.3)	19 (26.4)	
Homemaker	15 (21.7)	10 (14.5)	7 (9.7)	
Occupational activity				
Sedentary	38 (55.1)	38 (55.1)	38 (52.8)	0.985
Moderate	19 (27.5)	19 (27.5)	19 (26.4)	
Physically active	12 (17.4)	12 (17.4)	15 (20.8)	
Work environment				
Indoor	45 (65.2)	44 (63.8)	46 (63.9)	0.961
Outdoor	14 (20.3)	12 (17.4)	13 (18.1)	
Mixed	10 (14.5)	13 (18.8)	13 (18.1)	
Prolonged desk/screen-related neck flexion ≥ 6 h/day				
No	27 (39.1)	28 (40.6)	29 (40.3)	0.983
Yes	42 (60.9)	41 (59.4)	43 (59.7)	
Exercise level				
None/low	43 (62.3)	41 (59.4)	45 (62.5)	0.993
1–2 days/week	20 (29.0)	21 (30.4)	21 (29.2)	
≥ 3 days/week	6 (8.7)	7 (10.1)	6 (8.3)	
Blood sampling season				
Winter	29 (42.0)	28 (40.6)	32 (44.4)	0.985
Spring	15 (21.7)	17 (24.6)	18 (25.0)	
Summer	15 (21.7)	15 (21.7)	15 (20.8)	
Autumn	10 (14.5)	9 (13.0)	7 (9.7)	

Values are presented as median (Q1-Q3) or *n* (%). Age and BMI were compared using the Kruskal–Wallis test. Categorical variables were compared using Pearson’s chi-square test. *25*(*OH*)*D *25-Hydroxyvitamin D, *BMI* Body mass index

Table 2 presents the clinical scores before and after treatment, categorized by serum 25(OH)D levels. At baseline, statistically significant differences were evident among the groups across all clinical scales. Patients with vitamin D deficiency exhibited the highest scores on the NDI, VDI-QOL, VDI-SS, and DHI scales, whereas those with sufficient vitamin D levels demonstrated the lowest baseline symptom and disability scores. Following treatment, significant intergroup differences persisted for the NDI, VDI-QOL, and VDI-SS scores. Conversely, post-treatment DHI scores were similar across the three vitamin D groups, indicating that the perceived dizziness-related handicap reached comparable levels after treatment, regardless of baseline vitamin D status.

**Table 2:** Pre- and post-treatment NDI, VDI-QOL, VDI-SS, and DHI scores according to serum 25(OH)D categories

Variable	< 20 ng/mL (*n* = 69)	20–29 ng/mL (*n* = 69)	≥ 30 ng/mL (*n* = 72)	*P* value
Pre-treatment NDI	21 (20–23)ᵃ	16 (14–17)ᵇ	9 (8–11)ᶜ	< 0.001
Post-treatment NDI	7 (5–8)ᵃ	4 (3–4)ᵇ	3 (2–3)ᶜ	< 0.001
Pre-treatment VDI-QOL	73 (70–78)ᵃ	57 (54–60)ᵇ	41.5 (40–45)ᶜ	< 0.001
Post-treatment VDI-QOL	18 (16–19)ᵃ	13 (11–15)ᵇ	8 (8–9)ᶜ	< 0.001
Pre-treatment VDI-SS	52 (50–58)ᵃ	43 (40–45)ᵇ	37 (35–38)ᶜ	< 0.001
Post-treatment VDI-SS	9 (8–12)ᵃ	8 (7–9)ᵇ	10 (8–13)ᵃ	< 0.001
Pre-treatment DHI	57 (55–60)ᵃ	48 (46–50)ᵇ	44 (42–45)ᶜ	< 0.001
Post-treatment DHI	19 (17–19)ᵃ	19 (18–20)ᵃ	19 (18–20)ᵃ	0.108

Values are presented as median (Q1-Q3). *P* values were obtained using the Kruskal–Wallis test. Superscript letters (ᵃ, ᵇ, ᶜ) indicate statistically significant pairwise differences between groups after post hoc analysis; groups sharing the same letter are not significantly different. *NDI* Neck Disability Index, *VDI-QOL* Vertigo/Dizziness-Related Quality of Life subscale of the Vertigo Dizziness Imbalance questionnaire, *VDI-SS* Symptom Severity subscale of the Vertigo Dizziness Imbalance questionnaire, *DHI* Dizziness Handicap Inventory, *25*(*OH*)*D* 25-hydroxyvitamin D

**Fig. 1:**
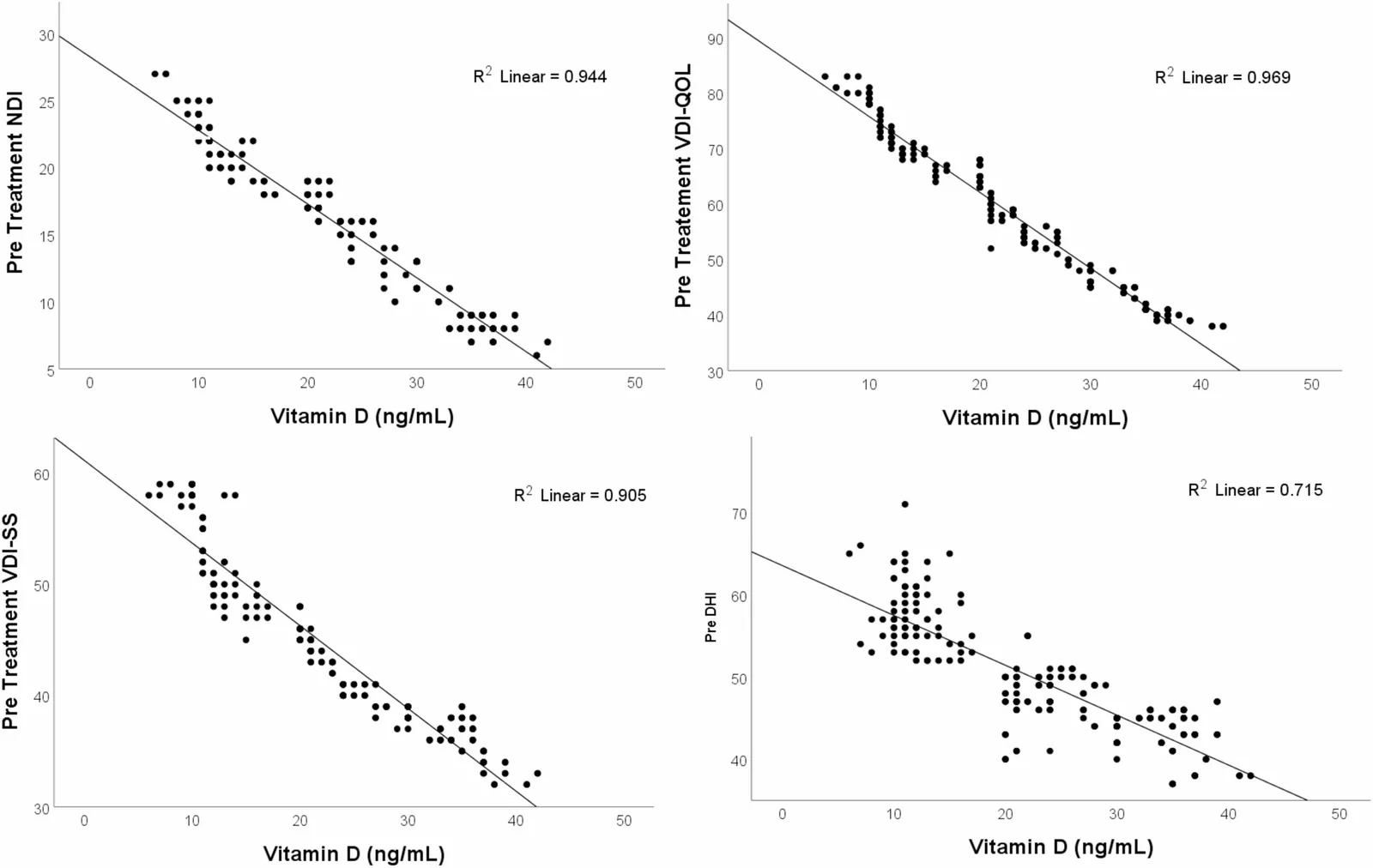
Association between serum vitamin D levels and baseline clinical severity. Scatter plots showing the relationships between baseline serum 25-hydroxyvitamin D [25(OH)D] levels and pretreatment Neck Disability Index (NDI), Dizziness Handicap Inventory (DHI), Vertigo Dizziness Imbalance–Symptom Scale (VDI-SS), and Vertigo Dizziness Imbalance–Quality of Life (VDI-QOL) scores. Solid lines represent fitted regression lines, and coefficients of determination (R²) are displayed for each plot

The association between serum 25(OH)D concentrations and pre-treatment clinical assessment scores is depicted in Fig. 1. Spearman correlation analysis revealed significant negative correlations between serum 25(OH)D levels and baseline NDI (rho = -0.975, *p* < 0.001), VDI-QOL (rho = -0.992, *p* < 0.001), VDI-SS (rho = -0.978, *p* < 0.001), and DHI (rho = -0.847, *p* < 0.001). Consequently, lower serum vitamin D levels were correlated with increased baseline symptom severity, greater disability, and diminished dizziness-related quality of life. The complete correlation matrix is available in Supplementary Table S1.

**Fig. 2:**
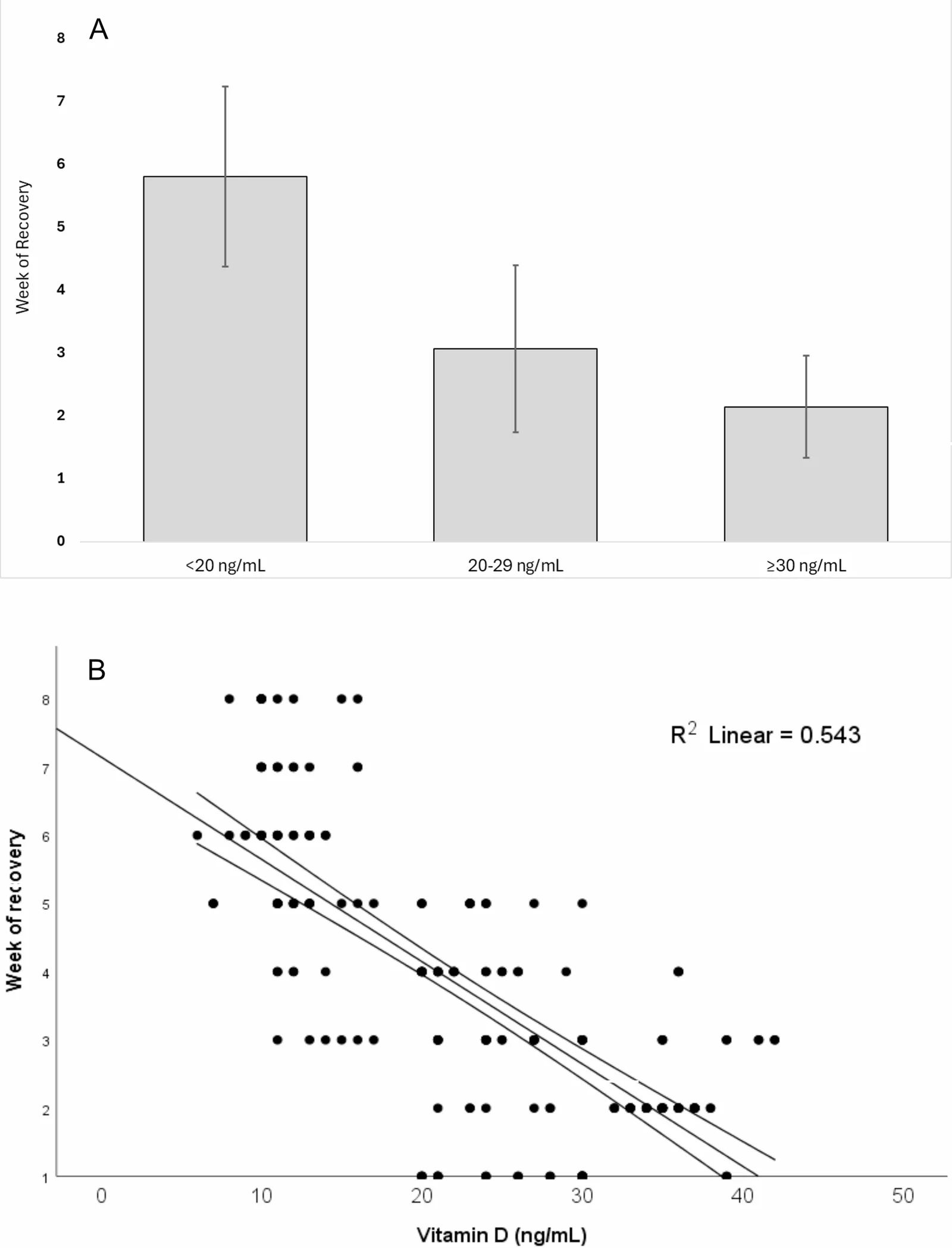
Association between serum vitamin D levels and recovery duration. **A** Bar graph showing recovery duration according to baseline serum 25-hydroxyvitamin D [25(OH)D] categories (< 20 ng/mL, 20–29 ng/mL, and ≥ 30 ng/mL). Error bars indicate variability. **B** Scatter plot demonstrating the relationship between baseline serum 25(OH)D levels and recovery duration expressed in weeks. Each point represents an individual patient. The fitted regression line illustrates an inverse association between serum vitamin D levels and time to clinical recovery

There was a significant variation in symptomatic recovery time among the three vitamin D groups, as determined by the Kruskal–Wallis test (*p* < 0.001). Subsequent post hoc analyses revealed that patients in the vitamin D deficiency group experienced a significantly delayed recovery compared to those in the insufficiency and sufficiency groups. An inverse correlation was identified between serum 25(OH)D levels and the week of recovery (rho = -0.77, *p* < 0.001; Fig. 2). In the multivariable linear analysis, serum 25(OH)D level was independently associated with recovery duration (β = -0.070 weeks/ng/mL; *p* = 0.002). When comparing early versus delayed recovery, the sex distribution was similar between the groups (*p* = 0.41), and there was no significant difference in mean age (early recovery: 30 ± 4 years; delayed recovery: 29 ± 3 years; *p* = 0.465). Serum vitamin D levels were higher in the early recovery group compared to the delayed recovery group (24 ± 10 vs. 21 ± 9 ng/mL; *p* = 0.04).

A multivariable binary logistic regression model was utilized to investigate the factors linked to delayed recovery, accounting for demographic, anthropometric, occupational, and lifestyle covariates. The comprehensive model included serum 25(OH)D level, age, sex, BMI, occupation category, occupational activity level, work environment, prolonged desk/screen-related neck-flexion exposure, and exercise level. In the adjusted analysis, a lower serum 25(OH)D level was significantly associated with delayed recovery (adjusted OR = 0.679 per 1 ng/mL increase, 95% CI: 0.604–0.765; *p* < 0.001). Age also exhibited a modest inverse relationship with delayed recovery (adjusted OR = 0.823 per year, 95% CI: 0.691–0.980; *p* = 0.029). Office/desk work was associated with delayed recovery compared to student status, although the wide confidence interval (adjusted OR = 31.716, 95% CI: 1.872-537.266; *p* = 0.017) suggests imprecision, warranting cautious interpretation. Other factors such as sex, BMI, occupational activity level, work environment, prolonged desk/screen-related neck-flexion exposure, and exercise level did not reach statistical significance in the adjusted model. These results underscore an inverse association between serum 25(OH)D level and recovery duration; however, due to the observational nature of the study, causality cannot be inferred.

**Fig. 3:**
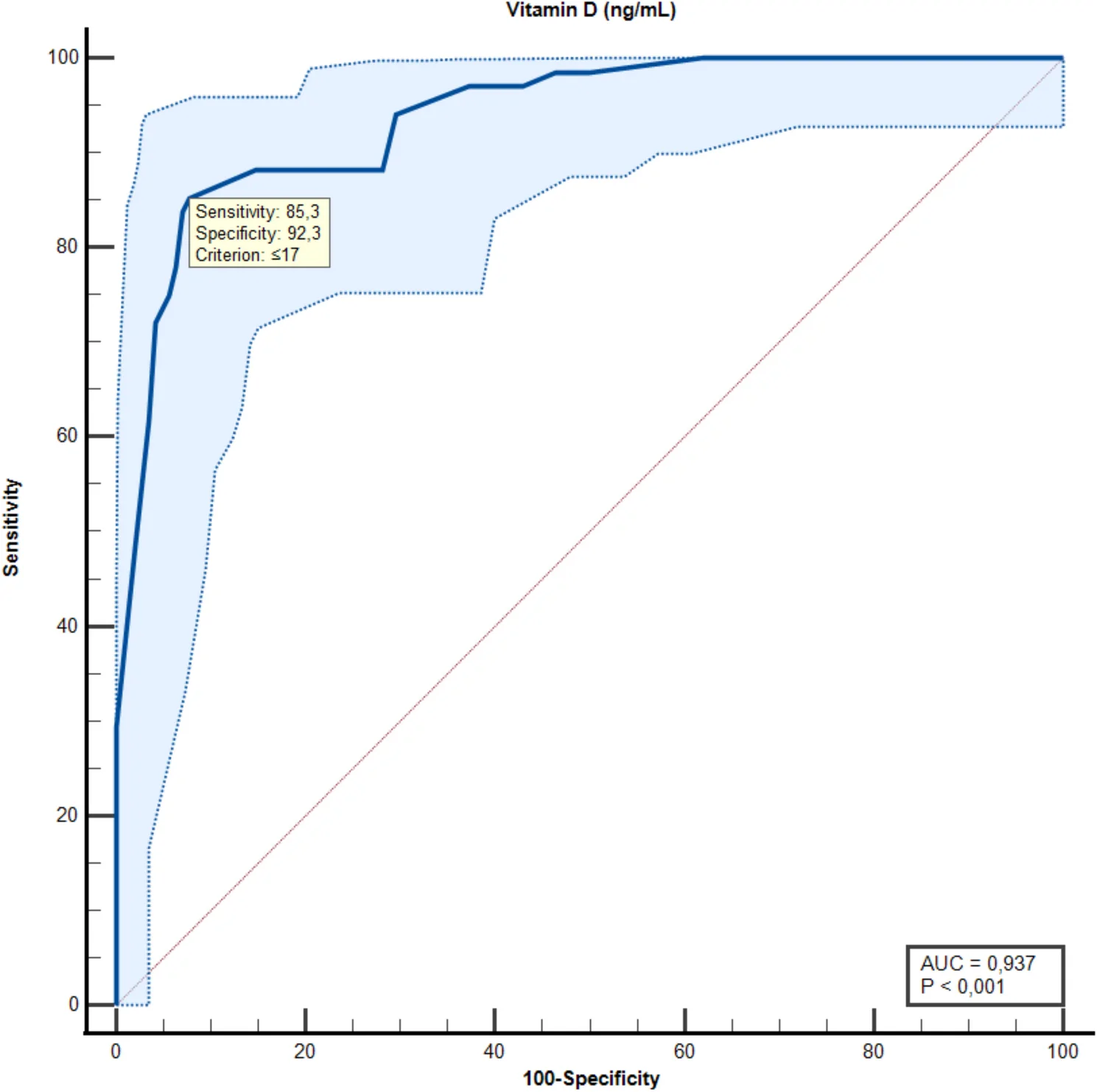
Receiver operating characteristic analysis for delayed recovery. Receiver Operating Characteristic (ROC) curve illustrating the ability of baseline serum 25-hydroxyvitamin D [25(OH)D] levels to discriminate between early recovery (≤ 4 weeks) and delayed recovery (≥ 5 weeks). The Area Under the Curve (AUC) represents overall discriminative performance. The optimal cutoff value was determined using the Youden index, with corresponding sensitivity and specificity values

Receiver operating characteristic (ROC) curve analysis was performed to assess the discriminatory power of serum vitamin D levels in distinguishing between early and delayed recovery groups. As illustrated in Fig. 3, the area under the curve (AUC) for serum vitamin D was 0.937, indicating excellent discriminatory capability (SE = 0.0168, 95% CI: 0.895–0.966, *p* < 0.0001). The optimal exploratory cut-off value, determined by the maximum Youden index, was ≤ 17 ng/mL (J = 0.7755). At this threshold, sensitivity was 85.29% (95% CI: 74.6–92.7), and specificity was 92.25% (95% CI: 86.6–96.1). These findings suggest a strong association between low serum vitamin D levels, particularly those ≤ 17 ng/mL, and delayed recovery in this cohort. As the ROC threshold was derived from the same study sample, it should be considered exploratory and requires external validation.

**Fig. 4:**
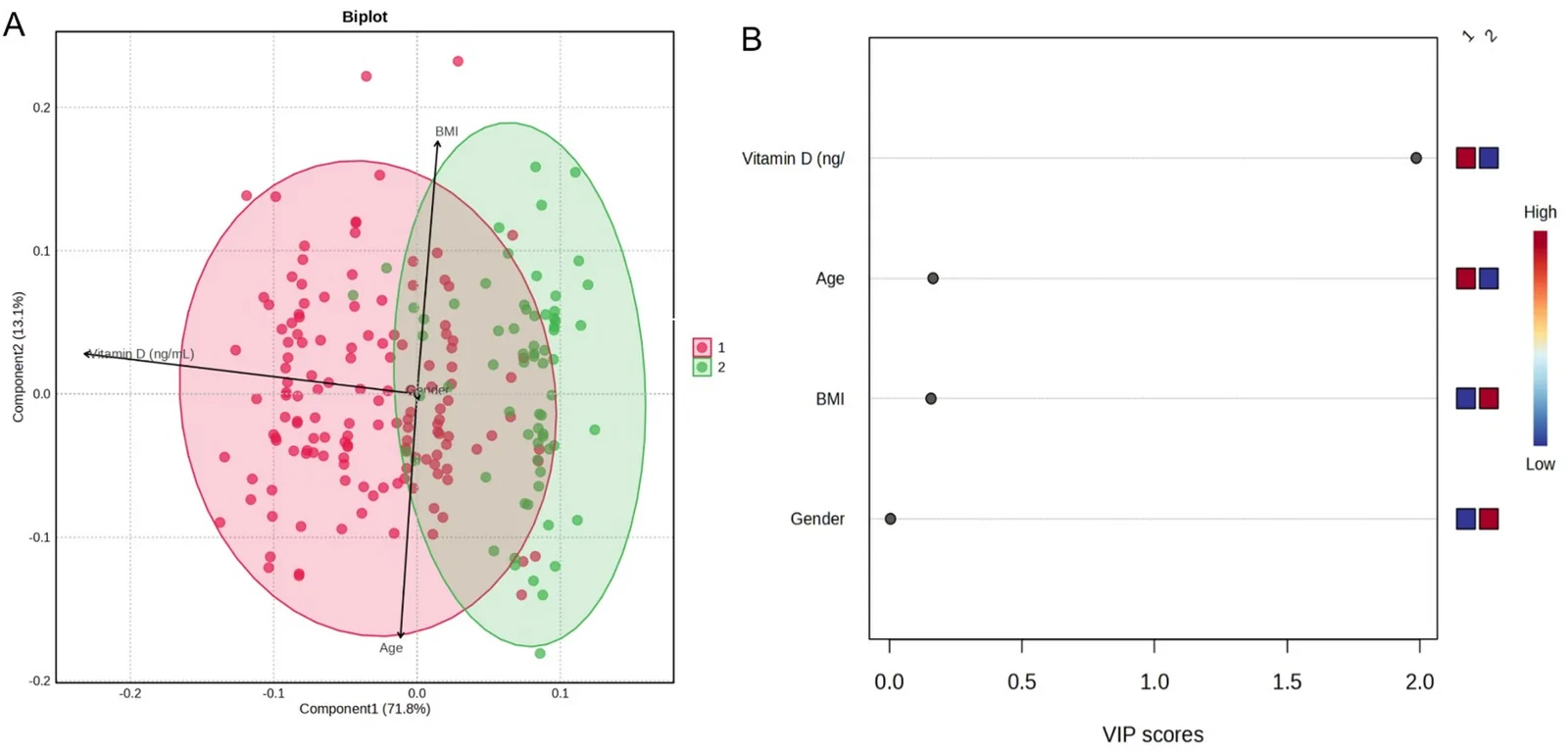
Multivariate discrimination of early and delayed recovery using partial least squares discriminant analysis. **A** Partial Least Squares Discriminant Analysis (PLS-DA) biplot showing the distribution of patients according to recovery status, defined as early recovery ≤ 4 weeks and delayed recovery ≥ 5 weeks, based on serum 25(OH)D level, age, sex, and Body Mass Index (BMI). The first two latent components explained 84.9% of the total variance in the model, with Component 1 accounting for 71.8% and Component 2 accounting for 13.1%. **B** Variable Importance in Projection (VIP) scores showing the relative contribution of each variable to group discrimination. Serum 25(OH)D level had the highest VIP score and was the principal variable contributing to group separation, whereas age, BMI, and sex contributed less to the discrimination pattern

Lastly, exploratory partial least squares discriminant analysis (PLS-DA) was conducted to evaluate the combined influence of serum 25(OH)D level, age, sex, and BMI on recovery status. The first two latent components accounted for 84.9% of the total variance in the model, with Component 1 explaining 71.8% and Component 2 explaining 13.1% (Fig. 4). Partial separation between early and delayed recovery groups was primarily observed along Component 1. Variable importance in projection (VIP) analysis indicated that serum 25(OH)D level had the highest VIP score, serving as the primary contributor to group separation, while age, BMI, and sex had lesser contributions. These findings should be regarded as exploratory and hypothesis-generating.

## Discussion

In this prospective observational study, lower baseline serum 25-hydroxyvitamin D [25(OH)D] levels were correlated with increased baseline clinical severity and an extended recovery period in patients with CD. This correlation was consistently observed across measures of symptom severity, neck-related disability, dizziness-related handicap, and dizziness-related quality of life. Serum 25(OH)D also demonstrated strong discriminatory capability for predicting delayed recovery in the ROC analysis and remained associated with delayed recovery after adjusting for available demographic, anthropometric, occupational, and lifestyle covariates. Collectively, these findings underscore the clinical significance of vitamin D status in this patient population; however, they should be interpreted as associative and hypothesis-generating rather than causal.

The current findings should be interpreted within the diagnostic complexity of CD. CD remains a challenging and somewhat controversial clinical entity due to the absence of a single definitive diagnostic test, necessitating the careful exclusion of vestibular, neurological, vascular, and systemic causes of dizziness [1, 2]. In this study, the diagnosis was corroborated by clinical examination, cervical findings, and supportive cervical tests, while alternative causes were excluded through audiovestibular, neurological, vascular, and imaging-based assessments. Consequently, the observed association between vitamin D levels and recovery should not be construed as evidence of a specific diagnostic mechanism for CD. Instead, it indicates that vitamin D deficiency may be a clinically relevant factor associated with symptom burden and recovery in a carefully selected CD cohort.

Previous research has established a correlation between vitamin D deficiency and benign paroxysmal positional vertigo (BPPV), with lower levels of vitamin D being linked to the onset, recurrence, persistence, and a less favorable clinical progression of the disease [19, 20]. Our findings expand upon this clinical observation by applying it to a different dizziness phenotype, although the underlying mechanisms are likely distinct. In the context of BPPV, vitamin D is typically discussed in relation to calcium metabolism and the integrity of otoconia. Conversely, cervical dizziness (CD) is believed to involve altered cervical proprioceptive input and impaired integration of cervical, vestibular, and visual signals [1, 11]. Therefore, our findings do not imply that CD and BPPV share the same pathophysiological pathway. Rather, they suggest the possibility that vitamin D deficiency may affect different dizziness syndromes through condition-specific biological mechanisms.

A plausible explanation for the current findings is the association between vitamin D deficiency and musculoskeletal dysfunction [21, 22]. Vitamin D deficiency has been linked to muscle pain, weakness, fatigue, impaired postural stability, and diminished neuromuscular control. These factors are clinically significant in CD, as cervical muscles, particularly the deep cervical flexor and extensor systems, play a role in both mechanical stabilization and proprioceptive afferent input [23, 24]. Dysfunction in these muscle groups may lead to increased abnormal cervical afferent signaling and impaired sensorimotor integration. In this context, lower vitamin D levels may correlate with more pronounced baseline symptoms and slower recovery due to effects on muscle performance, pain sensitivity, postural control, and proprioceptive regulation. Potential connections with neuroinflammation and neural adaptation may also be pertinent, although these mechanisms remain speculative in the present study [25].

The questionnaire results reveal clinically relevant insights. Initial assessments using the Neck Disability Index (NDI), Dizziness Handicap Inventory (DHI), Vestibular Disorders Activities of Daily Living Scale (VDI-SS), and Vestibular Disorders Quality of Life (VDI-QOL) indicated poorer outcomes in patients with lower vitamin D levels. This finding suggests a correlation between vitamin D deficiency and increased neck-related disability, as well as heightened perceived dizziness and diminished quality of life. Following treatment, DHI scores became more uniform across the vitamin D groups, while differences in NDI, VDI-SS, and VDI-QOL scores persisted. This pattern may suggest that a standardized multimodal rehabilitation program can effectively alleviate dizziness-related handicap, whereas ongoing neck-related disability and quality-of-life issues related to dizziness may be more closely tied to the initial musculoskeletal condition. This interpretation supports the clinical perspective that cervicogenic dizziness (CD) encompasses not only vestibular symptoms but also cervical musculoskeletal and proprioceptive dysfunction.

The findings regarding recovery duration hold significant clinical implications. All patients were subjected to a uniform multimodal rehabilitation protocol, encompassing manual therapy, deep cervical flexor strengthening, proprioceptive training, balance exercises, stretching, ergonomic education, and home exercises. Notwithstanding this standardized regimen, patients exhibiting lower baseline vitamin D levels experienced a protracted recovery period. This observation implies that vitamin D status may serve as an indicator of delayed functional recovery in CD. However, given that patients with deficient or insufficient vitamin D levels also received supplementation as part of routine care, the current study is unable to ascertain whether the observed association is attributable to baseline biological status, response to supplementation, variations in musculoskeletal reserve, or unmeasured lifestyle factors.

The incorporation of demographic, anthropometric, occupational, lifestyle, and seasonal characteristics enhances the clinical interpretation of the current study. Variables such as age, sex, BMI, occupation category, occupational activity level, work environment, prolonged desk/screen-related neck-flexion exposure, exercise level, and the season of blood sampling were comparable across the vitamin D groups. In the adjusted delayed-recovery model, most occupational and lifestyle variables did not exhibit significant associations with delayed recovery. Although one occupational category achieved statistical significance, the broad confidence interval suggests imprecision, likely due to the small size of certain occupational subgroups. Consequently, this finding should be interpreted with caution. The primary adjusted association persisted between lower serum 25(OH)D levels and delayed recovery.

The results from the ROC and PLS-DA analyses warrant careful interpretation [26]. The ROC analysis demonstrated a high level of discriminatory capability for delayed recovery, identifying an exploratory threshold of ≤ 17 ng/mL. However, this threshold was established using the same cohort and lacks external validation. Therefore, it should not be considered a definitive clinical cutoff or used as an independent decision-making criterion. Similarly, the PLS-DA analysis suggested that serum 25(OH)D played a more significant role in distinguishing recovery status compared to age, sex, or BMI, although this multivariate analysis remains exploratory. These analyses should be viewed as reinforcing the internal consistency of the observed association and as a basis for generating hypotheses for future validation studies.

From a practical perspective, the current findings indicate that serum vitamin D levels may warrant consideration during the clinical evaluation of patients with cervical dystonia (CD), particularly in those exhibiting significant neck pain, elevated baseline disability scores, or delayed recovery. Nevertheless, these results should not be construed as evidence that vitamin D deficiency is the primary etiology of CD or that supplementation alone constitutes adequate treatment. The primary management of CD should continue to emphasize precise differential diagnosis, the exclusion of serious vestibular or neurological causes, and tailored cervical rehabilitation. Vitamin D assessment may be regarded as an adjunctive clinical factor rather than a substitute for established evaluation and treatment protocols.

Several limitations of this study should be acknowledged, including its observational single-center design, the absence of a control group, incomplete assessment of lifestyle factors and biomarkers, and the lack of objective measures for cervical function or inter-rater reliability. Given that recovery was clinically defined and the ROC cutoff was exploratory, these findings necessitate external validation through controlled studies.

## Conclusion

In conclusion, lower serum 25(OH)D levels were associated with greater baseline clinical severity and a longer recovery course in patients with CD. Vitamin D status may therefore be worth considering as part of the clinical context in this population. Further prospective controlled studies are needed to clarify the biological and clinical significance of this association.

## Supplementary Information

Supplementary Material 1.

## Data Availability

The datasets generated and/or analysed during the current study are available from the corresponding author on reasonable request.
