# Supplementary material for: Association of serum vitamin D levels with clinical severity and recovery duration in cervical dizziness
Source: BMC Musculoskelet Disord. 2026 Jun 23;27:757. doi: 10.1186/s12891-026-10119-1 (PMC13543346; doi:10.1186/s12891-026-10119-1)
Supplement: Supplementary file 1 [file 12891_2026_10119_MOESM1_ESM.docx]

Table s1: The all correlation between all variables

|  | | Age (year) | Time since onset of neck pain | | Pre Treatment NDI | | Post Treatment NDI | | Pre Treatement VDI-QOL | | Post Treatment VDI-QOL | | Pre Treatment VDI-SS | | Post Treatment VDI-SS | | Pre Fonctional | Pre EMOS | Pre Physical | | Post Fonctional | | Post EMOS | | Post Physical | | Pre DHI | | Post DHI | | Vitamin D (ng/mL) | |
| --- | --- | --- | --- | --- | --- | --- | --- | --- | --- | --- | --- | --- | --- | --- | --- | --- | --- | --- | --- | --- | --- | --- | --- | --- | --- | --- | --- | --- | --- | --- | --- | --- |
| Spearman's rho | Age (year) | Correlation Coefficient | | 1.000 | | .057 | | -.109 | | -.084 | | -.102 | | -.113 | | -.092 | .064 | -.014 | -.017 | -.093 | | -.052 | | -.081 | | -.002 | | -.081 | | -.127 | | .113 |
|  |  | Sig. (2-tailed) | | . | | .415 | | .114 | | .223 | | .140 | | .103 | | .182 | .353 | .839 | .810 | .181 | | .454 | | .241 | | .980 | | .245 | | .066 | | .103 |
|  |  | N | | 210 | | 210 | | 210 | | 210 | | 210 | | 210 | | 210 | 210 | 210 | 210 | 210 | | 210 | | 210 | | 210 | | 210 | | 210 | | 210 |
|  | Time since onset of neck pain | Correlation Coefficient | | .057 | | 1.000 | | -.111 | | -.145^*^ | | -.121 | | -.090 | | -.096 | .076 | -.051 | -.158^*^ | -.035 | | -.054 | | .038 | | .096 | | -.181^**^ | | .071 | | .117 |
|  |  | Sig. (2-tailed) | | .415 | | . | | .107 | | .036 | | .081 | | .193 | | .165 | .276 | .465 | .022 | .615 | | .433 | | .580 | | .167 | | .009 | | .308 | | .092 |
|  |  | N | | 210 | | 210 | | 210 | | 210 | | 210 | | 210 | | 210 | 210 | 210 | 210 | 210 | | 210 | | 210 | | 210 | | 210 | | 210 | | 210 |
|  | Pre Treatment NDI | Correlation Coefficient | | -.109 | | -.111 | | 1.000 | | .696^**^ | | .964^**^ | | .867^**^ | | .961^**^ | -.014 | .606^**^ | .494^**^ | .394^**^ | | .012 | | -.109 | | -.161^*^ | | .844^**^ | | -.124 | | -.975^**^ |
|  |  | Sig. (2-tailed) | | .114 | | .107 | | . | | .000 | | .000 | | .000 | | .000 | .838 | .000 | .000 | .000 | | .860 | | .115 | | .020 | | .000 | | .073 | | .000 |
|  |  | N | | 210 | | 210 | | 210 | | 210 | | 210 | | 210 | | 210 | 210 | 210 | 210 | 210 | | 210 | | 210 | | 210 | | 210 | | 210 | | 210 |
|  | Post Treatment NDI | Correlation Coefficient | | -.084 | | -.145^*^ | | .696^**^ | | 1.000 | | .706^**^ | | .618^**^ | | .684^**^ | .032 | .463^**^ | .413^**^ | .346^**^ | | .134 | | -.101 | | -.199^**^ | | .690^**^ | | -.072 | | -.706^**^ |
|  |  | Sig. (2-tailed) | | .223 | | .036 | | .000 | | . | | .000 | | .000 | | .000 | .645 | .000 | .000 | .000 | | .053 | | .145 | | .004 | | .000 | | .301 | | .000 |
|  |  | N | | 210 | | 210 | | 210 | | 210 | | 210 | | 210 | | 210 | 210 | 210 | 210 | 210 | | 210 | | 210 | | 210 | | 210 | | 210 | | 210 |
|  | Pre Treatement VDI-QOL | Correlation Coefficient | | -.102 | | -.121 | | .964^**^ | | .706^**^ | | 1.000 | | .886^**^ | | .968^**^ | -.035 | .569^**^ | .491^**^ | .387^**^ | | -.003 | | -.099 | | -.193^**^ | | .825^**^ | | -.122 | | -.992^**^ |
|  |  | Sig. (2-tailed) | | .140 | | .081 | | .000 | | .000 | | . | | .000 | | .000 | .618 | .000 | .000 | .000 | | .960 | | .151 | | .005 | | .000 | | .079 | | .000 |
|  |  | N | | 210 | | 210 | | 210 | | 210 | | 210 | | 210 | | 210 | 210 | 210 | 210 | 210 | | 210 | | 210 | | 210 | | 210 | | 210 | | 210 |
|  | Post Treatment VDI-QOL | Correlation Coefficient | | -.113 | | -.090 | | .867^**^ | | .618^**^ | | .886^**^ | | 1.000 | | .880^**^ | -.105 | .533^**^ | .404^**^ | .349^**^ | | -.019 | | -.072 | | -.159^*^ | | .744^**^ | | -.032 | | -.887^**^ |
|  |  | Sig. (2-tailed) | | .103 | | .193 | | .000 | | .000 | | .000 | | . | | .000 | .129 | .000 | .000 | .000 | | .783 | | .297 | | .021 | | .000 | | .647 | | .000 |
|  |  | N | | 210 | | 210 | | 210 | | 210 | | 210 | | 210 | | 210 | 210 | 210 | 210 | 210 | | 210 | | 210 | | 210 | | 210 | | 210 | | 210 |
|  | Pre Treatment VDI-SS | Correlation Coefficient | | -.092 | | -.096 | | .961^**^ | | .684^**^ | | .968^**^ | | .880^**^ | | 1.000 | -.033 | .580^**^ | .512^**^ | .385^**^ | | -.011 | | -.055 | | -.139^*^ | | .837^**^ | | -.092 | | -.978^**^ |
|  |  | Sig. (2-tailed) | | .182 | | .165 | | .000 | | .000 | | .000 | | .000 | | . | .638 | .000 | .000 | .000 | | .877 | | .428 | | .044 | | .000 | | .183 | | .000 |
|  |  | N | | 210 | | 210 | | 210 | | 210 | | 210 | | 210 | | 210 | 210 | 210 | 210 | 210 | | 210 | | 210 | | 210 | | 210 | | 210 | | 210 |
|  | Post Treatment VDI-SS | Correlation Coefficient | | .064 | | .076 | | -.014 | | .032 | | -.035 | | -.105 | | -.033 | 1.000 | -.148^*^ | -.008 | -.118 | | .059 | | -.008 | | .112 | | -.144^*^ | | .003 | | .030 |
|  |  | Sig. (2-tailed) | | .353 | | .276 | | .838 | | .645 | | .618 | | .129 | | .638 | . | .032 | .904 | .089 | | .397 | | .912 | | .107 | | .038 | | .964 | | .665 |
|  |  | N | | 210 | | 210 | | 210 | | 210 | | 210 | | 210 | | 210 | 210 | 210 | 210 | 210 | | 210 | | 210 | | 210 | | 210 | | 210 | | 210 |
|  | Pre Fonctional | Correlation Coefficient | | -.014 | | -.051 | | .606^**^ | | .463^**^ | | .569^**^ | | .533^**^ | | .580^**^ | -.148^*^ | 1.000 | .204^**^ | .139^*^ | | -.013 | | -.025 | | -.240^**^ | | .712^**^ | | -.232^**^ | | -.591^**^ |
|  |  | Sig. (2-tailed) | | .839 | | .465 | | .000 | | .000 | | .000 | | .000 | | .000 | .032 | . | .003 | .044 | | .851 | | .718 | | .000 | | .000 | | .001 | | .000 |
|  |  | N | | 210 | | 210 | | 210 | | 210 | | 210 | | 210 | | 210 | 210 | 210 | 210 | 210 | | 210 | | 210 | | 210 | | 210 | | 210 | | 210 |
|  | Pre EMOS | Correlation Coefficient | | -.017 | | -.158^*^ | | .494^**^ | | .413^**^ | | .491^**^ | | .404^**^ | | .512^**^ | -.008 | .204^**^ | 1.000 | -.090 | | .023 | | -.004 | | -.093 | | .585^**^ | | -.018 | | -.515^**^ |
|  |  | Sig. (2-tailed) | | .810 | | .022 | | .000 | | .000 | | .000 | | .000 | | .000 | .904 | .003 | . | .191 | | .745 | | .958 | | .180 | | .000 | | .794 | | .000 |
|  |  | N | | 210 | | 210 | | 210 | | 210 | | 210 | | 210 | | 210 | 210 | 210 | 210 | 210 | | 210 | | 210 | | 210 | | 210 | | 210 | | 210 |
|  | Pre Physical | Correlation Coefficient | | -.093 | | -.035 | | .394^**^ | | .346^**^ | | .387^**^ | | .349^**^ | | .385^**^ | -.118 | .139^*^ | -.090 | 1.000 | | .044 | | -.066 | | .013 | | .498^**^ | | -.078 | | -.378^**^ |
|  |  | Sig. (2-tailed) | | .181 | | .615 | | .000 | | .000 | | .000 | | .000 | | .000 | .089 | .044 | .191 | . | | .529 | | .343 | | .847 | | .000 | | .259 | | .000 |
|  |  | N | | 210 | | 210 | | 210 | | 210 | | 210 | | 210 | | 210 | 210 | 210 | 210 | 210 | | 210 | | 210 | | 210 | | 210 | | 210 | | 210 |
|  | Post Fonctional | Correlation Coefficient | | -.052 | | -.054 | | .012 | | .134 | | -.003 | | -.019 | | -.011 | .059 | -.013 | .023 | .044 | | 1.000 | | .235^**^ | | -.075 | | .048 | | .374^**^ | | -.010 |
|  |  | Sig. (2-tailed) | | .454 | | .433 | | .860 | | .053 | | .960 | | .783 | | .877 | .397 | .851 | .745 | .529 | | . | | .001 | | .280 | | .493 | | .000 | | .890 |
|  |  | N | | 210 | | 210 | | 210 | | 210 | | 210 | | 210 | | 210 | 210 | 210 | 210 | 210 | | 210 | | 210 | | 210 | | 210 | | 210 | | 210 |
|  | Post EMOS | Correlation Coefficient | | -.081 | | .038 | | -.109 | | -.101 | | -.099 | | -.072 | | -.055 | -.008 | -.025 | -.004 | -.066 | | .235^**^ | | 1.000 | | .111 | | -.071 | | .534^**^ | | .083 |
|  |  | Sig. (2-tailed) | | .241 | | .580 | | .115 | | .145 | | .151 | | .297 | | .428 | .912 | .718 | .958 | .343 | | .001 | | . | | .109 | | .304 | | .000 | | .229 |
|  |  | N | | 210 | | 210 | | 210 | | 210 | | 210 | | 210 | | 210 | 210 | 210 | 210 | 210 | | 210 | | 210 | | 210 | | 210 | | 210 | | 210 |
|  | Post Physical | Correlation Coefficient | | -.002 | | .096 | | -.161^*^ | | -.199^**^ | | -.193^**^ | | -.159^*^ | | -.139^*^ | .112 | -.240^**^ | -.093 | .013 | | -.075 | | .111 | | 1.000 | | -.226^**^ | | .328^**^ | | .187^**^ |
|  |  | Sig. (2-tailed) | | .980 | | .167 | | .020 | | .004 | | .005 | | .021 | | .044 | .107 | .000 | .180 | .847 | | .280 | | .109 | | . | | .001 | | .000 | | .007 |
|  |  | N | | 210 | | 210 | | 210 | | 210 | | 210 | | 210 | | 210 | 210 | 210 | 210 | 210 | | 210 | | 210 | | 210 | | 210 | | 210 | | 210 |
|  | Pre DHI | Correlation Coefficient | | -.081 | | -.181^**^ | | .844^**^ | | .690^**^ | | .825^**^ | | .744^**^ | | .837^**^ | -.144^*^ | .712^**^ | .585^**^ | .498^**^ | | .048 | | -.071 | | -.226^**^ | | 1.000 | | -.163^*^ | | -.847^**^ |
|  |  | Sig. (2-tailed) | | .245 | | .009 | | .000 | | .000 | | .000 | | .000 | | .000 | .038 | .000 | .000 | .000 | | .493 | | .304 | | .001 | | . | | .018 | | .000 |
|  |  | N | | 210 | | 210 | | 210 | | 210 | | 210 | | 210 | | 210 | 210 | 210 | 210 | 210 | | 210 | | 210 | | 210 | | 210 | | 210 | | 210 |
|  | Post DHI | Correlation Coefficient | | -.127 | | .071 | | -.124 | | -.072 | | -.122 | | -.032 | | -.092 | .003 | -.232^**^ | -.018 | -.078 | | .374^**^ | | .534^**^ | | .328^**^ | | -.163^*^ | | 1.000 | | .112 |
|  |  | Sig. (2-tailed) | | .066 | | .308 | | .073 | | .301 | | .079 | | .647 | | .183 | .964 | .001 | .794 | .259 | | .000 | | .000 | | .000 | | .018 | | . | | .107 |
|  |  | N | | 210 | | 210 | | 210 | | 210 | | 210 | | 210 | | 210 | 210 | 210 | 210 | 210 | | 210 | | 210 | | 210 | | 210 | | 210 | | 210 |
|  | Vitamin D (ng/mL) | Correlation Coefficient | | .113 | | .117 | | -.975^**^ | | -.706^**^ | | -.992^**^ | | -.887^**^ | | -.978^**^ | .030 | -.591^**^ | -.515^**^ | -.378^**^ | | -.010 | | .083 | | .187^**^ | | -.847^**^ | | .112 | | 1.000 |
|  |  | Sig. (2-tailed) | | .103 | | .092 | | .000 | | .000 | | .000 | | .000 | | .000 | .665 | .000 | .000 | .000 | | .890 | | .229 | | .007 | | .000 | | .107 | | . |
|  |  | N | | 210 | | 210 | | 210 | | 210 | | 210 | | 210 | | 210 | 210 | 210 | 210 | 210 | | 210 | | 210 | | 210 | | 210 | | 210 | | 210 |
